# Flexural Stiffness and Force Retention of Six 3D-Printed Thermoplastics for Orthodontic Appliances: An In Vitro Screening Study

**DOI:** 10.3390/dj14090565

**Published:** 2026-09-04

**Authors:** Marco Serafin, Marina Borgese, Elisa Boccalari, Gilberto Binda, Mario Raspanti, Piero Antonio Zecca

**Affiliations:** 1 Department of Biomedical Sciences for Health, University of Milan, 20133 Milan, Italy; 2 Department of Medicine and Innovative Technology, University of Insubria, 21100 Varese, Italy; 3 Department of Theoretical and Applied Sciences, University of Insubria, 21100 Varese, Italy; 4 Norwegian Institute for Water Research (NIVA), 0579 Oslo, Norway

**Keywords:** additive manufacturing, fused deposition modelling, selective laser sintering, thermoplastic polymers, orthodontic appliances, three-point bending, stress relaxation, flexural modulus, FTIR spectroscopy

## Abstract

**Background/Objectives:** Directly printed orthodontic appliances rely mostly on vat-photopolymerisation resins; pre-polymerised thermoplastics processed by fused deposition modelling (FDM) and selective laser sintering (SLS) are a less-explored, single-component route, but are poorly characterised. This study compared six thermoplastic material–process systems, supplied for biomedical or dental use, using a single intraoral-simulating protocol. **Methods:** Thin specimens (25 × 5 × 0.6 mm) of polyethylene terephthalate glycol (PETG), polycarbonate (PC), polyamide (PA), polyether-ether-ketone (PEEK) and a thermoplastic copolyester elastomer (TPC) printed by FDM, and of polyamide 12 (PA12) by SLS, underwent three-point bending and 10 min stress relaxation at 37 °C in artificial saliva (*n* = 5). Differences were assessed using the Kruskal–Wallis test with Dunn post hoc comparisons (Holm correction), and Fourier-transform infrared (FTIR) spectroscopy was used to compare virgin and printed materials for the five FDM polymers. **Results:** Apparent flexural moduli were 989 (PA12), 750 (PC), 687 (PEEK), 509 (PETG), 85 (PA) and 32 MPa (TPC), a very large material effect (ε^2^ = 0.95), with post hoc comparisons separating only the extremes of that range. Relaxation over 10 min ranged from 2.6% (PC) to 22.1% (PA12) among the five materials in which it could be quantified, with TPC falling below the load-cell resolution; expressed as force, PA12 lost the most (0.86 N) and yet still delivered 3.01 N, which was above the initial force of PEEK, PETG, PA and TPC, though not separable from that of PC, which retained 97.4% of its own. Virgin and printed spectra were concordant (Pearson r ≥ 0.986, against ≤ 0.80 between different materials). **Conclusions:** The PA12/SLS system was the stiffest, followed by PC and PEEK, but, as the only laser-sintered material, its polymer and process effects cannot be separated. PETG and PC combine useful stiffness with accessible FDM; PA and TPC are far more compliant. These specimen-level findings provide a screening basis for selecting material–process systems for printed orthodontic appliances, preceding device-level validation.

## 1. Introduction

Laboratory and chairside 3D printing is now routine in dentistry, and orthodontics was among the first fields to adopt it. Most direct printing relies on vat-photopolymerisation resins, which reproduce fine detail but require wet post-processing and raise recognised concerns over residual monomer, leachate release and particulate shedding [1,2]. Pre-polymerised thermoplastics processed by fused deposition modelling (FDM) or selective laser sintering (SLS) are a far less-explored route: supplied as fully polymerised single-component materials, they print without wet post-processing and contain no photoinitiators or reactive monomers. However, their mechanical and viscoelastic behaviour in the thin sections relevant to orthodontic appliances remains poorly characterised.

Clear aligner therapy has transformed contemporary orthodontics by offering removable, nearly invisible appliances that deliver controlled tooth movement with high patient acceptance. Conventional aligners are thermoformed from thin sheets of polyethylene terephthalate glycol (PETG) or polyurethane over a 3D-printed model. During thermoforming, heating reduces material thickness and mechanical strength, and intraoral exposure to saliva and thermal fluctuations leads to water uptake, dimensional changes, and progressive softening [3]. The uniform thickness of commercial sheets also limits the ability to tailor force distribution across the aligner.

Direct 3D printing enables the manufacture of aligners and auxiliaries from a digital dental model, eliminating the need for a physical model and enabling precise control over geometry and thickness. Several additive manufacturing techniques are employed in dentistry: stereolithography (SLA), digital light processing (DLP), and liquid crystal display (LCD) cure photopolymer resins; fused deposition modelling extrudes thermoplastic filament; and selective laser sintering fuses powder with a laser to build strong parts. Mechanical performance depends on printer type, material composition, printing parameters, post-curing, and layer height. DLP and LCD technologies are popular for direct aligner fabrication, whereas FDM is less common because the layered deposition can yield a rougher surface and lower precision [3,4,5]. The properties that matter clinically for printable intraoral materials and the heterogeneity of the evidence regarding them have been reviewed critically [6]. Nevertheless, FDM is appealing due to its low cost and the broad range of available engineering thermoplastics, while SLS offers access to materials such as polyamide-12 (PA12).

The literature on FDM- and SLS-printed thermoplastics for orthodontic use is still nascent. Aligners produced by fused filament fabrication have been shown to deliver forces and moments that depend systematically on layer height [7], and thermoplastic filaments intended for intraoral use have been characterised after exposure to edible liquids [8]. In FDM studies, PETG reinforced with sepiolite nanoclay showed a 35.4% increase in tensile strength compared with injection-moulded PETG, as the needle-shaped fillers aligned along the printing direction during deposition [9]. Mechanical tests on FDM dental tray materials showed that PETG and recycled PLA exhibited flexural and tensile strengths comparable to conventional polymethyl methacrylate, with print orientation having only a modest effect on bond strength [10]. Although 3D printing has not yet been widely adopted for aligners, direct-printed aligners produced in a DLP-cured shape-memory polymer (Tera Harz TC-85) deliver forces within the biologically acceptable range [11]. These aligners are stiff at room temperature and soften at body temperature, improving deflection and force recovery [3,4]. Comparative analyses reveal that printing technology influences mechanical properties: LCD-printed aligners show higher hardness and indentation modulus than DLP-printed ones. Overall, directly printed aligners can match or exceed the stiffness of commercial thermoformed aligners, and blended printable resins have been benchmarked mechanically against nickel–titanium wire [12,13]. Finite-element simulations further indicate that stress distributions in DLP-printed resin aligners under compressive loading differ by only 0.2–7.7% from those in thermoformed PETG aligners [14]. Conversely, water-absorption studies show that thermoformed polyurethane/PETG laminates absorb more water and release it more quickly than 3D-printed resins [15].

A second reason to look at FDM filaments is compositional. Photopolymer resins are formulated with photoinitiators, reactive diluents, cross-linkers and UV stabilisers; printed dental resins can be cytotoxic in vitro even after post-curing and washing [16], leaching has been quantified by targeted chemical analysis [17], one printed aligner material was both cytotoxic and estrogenic [18], and printed aligners shed more micro- and nanoplastic than thermoformed devices [19], whose urethane particles shift macrophages towards pro-inflammatory phenotypes [20]. Medical-grade FDM filaments differ in where the biological evidence sits rather than in whether it exists. As pre-polymerised single-component polymers, their documentation is attached to the raw material. It follows standard panels: five of the six used here carry biological test data, cited against ISO 10993 [21] or USP Class VI panels in four cases, while the sixth is certified for food-contact use under FDA 21 CFR 177.1500 (Table 1). In a photopolymer, the material that reaches the patient does not exist until printing, so no raw-material data can be inherited. Qualifying a material biologically is the manufacturer’s task, not something a laboratory study repeats; what such a study can add is whether that qualification survives printing. That is a reason to investigate these filaments, not evidence that an appliance made from them is safer, since the documentation covers the material and not a finished device.

Despite growing interest in direct thermoplastic printing, no study has yet compared, under a common intraoral-simulating protocol, the flexural and viscoelastic behaviour of the thermoplastics supplied for biomedical use that are actually accessible through FDM and SLS. The present work addresses this gap by characterising six such materials (PETG, PC, PA, PEEK and TPC by FDM, and PA12 by SLS) using three-point bending with unloading–reloading cycles and short-term stress relaxation on thin specimens that reproduce the cross-section of printed aligners and auxiliaries. We hypothesised that the selected polymers would span a mechanical range wide enough to bracket the stiffness and force-retention requirements of orthodontic devices, and that the material–process systems would differ measurably in their time-dependent response. The clinical relevance lies in identifying which material–process systems warrant device-level evaluation, and for which class of appliance: aligners, rigid single-material components such as palatal bars and expansion auxiliaries, or components designed around large elastic deformation. These classes place different demands on stiffness and force retention, and the systems must remain printable on equipment available in laboratory or in-office settings.

## 2. Materials and Methods

### 2.1. Materials

**Figure 1:**
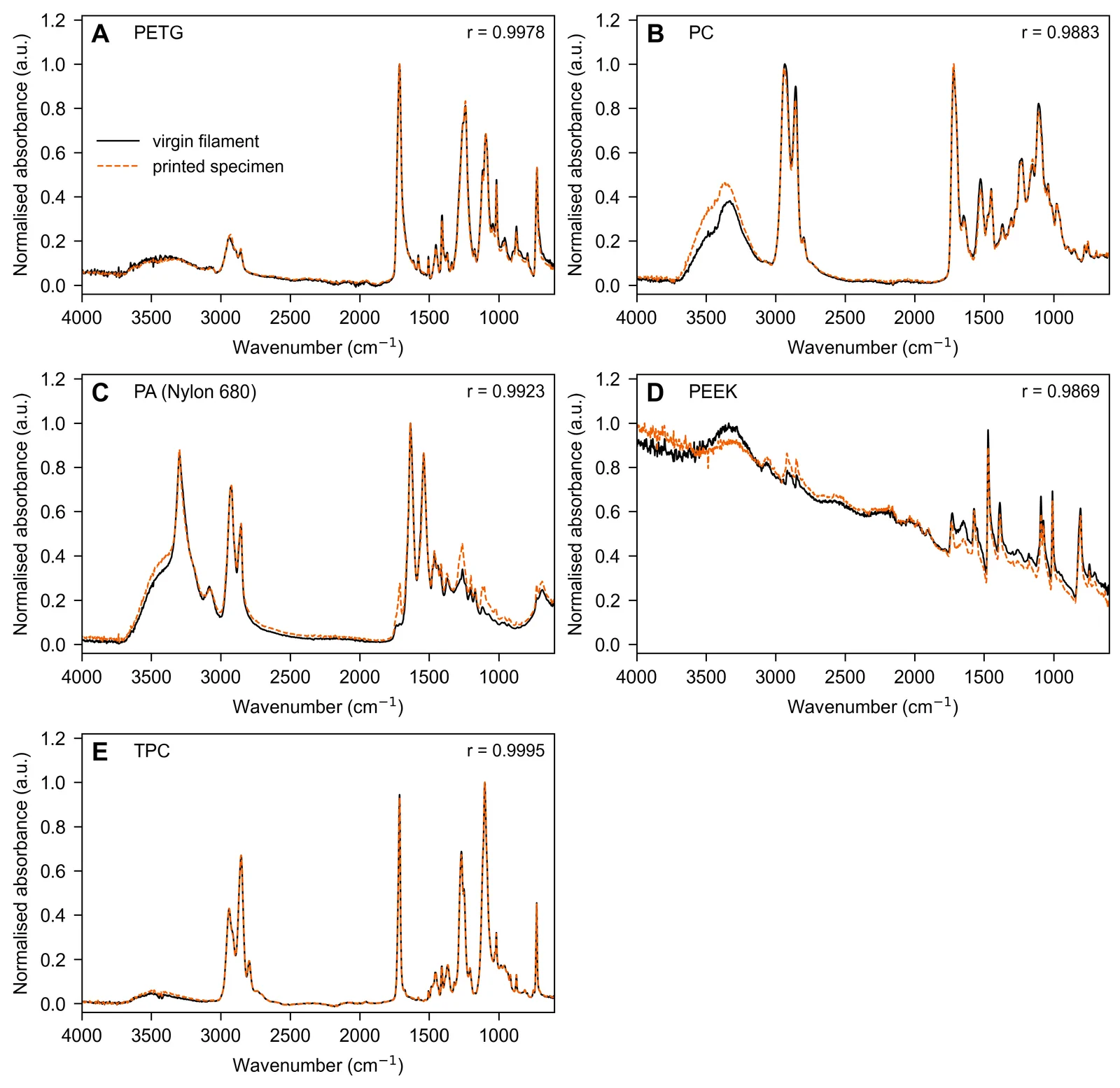
FTIR spectra of the FDM-processed thermoplastic materials. (**A**) PETG; (**B**) PC; (**C**) PA (Nylon 680); (**D**) PEEK; (**E**) TPC. In each panel, the virgin-filament spectrum (solid black) and the printed-specimen spectrum (dashed orange) are overlaid on the same scale; each spectrum is normalised to its own maximum absorbance, with no vertical offset and no baseline correction. r is the Pearson correlation between the two spectra of that material, computed point by point over 7052 points from 600.7 to 4000.1 cm^−1^. FTIR was not acquired on the SLS-processed PA12 (see Section 2.6).

The materials were selected based on the biological documentation provided by their manufacturers for biomedical or dental use; we performed no biological testing. The classification claimed by each supplier is given with the material in Table 1; the underlying test reports are held in the manufacturers’ technical documentation and were not independently verified by us. Five of the six materials (PETG, PC, PA, PEEK, and TPC) were processed by fused deposition modelling, while PA12 was produced by selective laser sintering. Only one laser-sintered material was included because PA12 is, at present, the only polymer for which selective laser sintering offers a route to orthodontic appliances: laser-sintered PEEK is not yet established for this use, and laser-sintered metals are far too rigid for appliances that must deflect and return. The single SLS entry is therefore a reflection of what is currently available, not a sampling choice. The BIOFLEX filament is supplied as a flexible copolyester-based elastomer, and its polymer class was verified on the virgin-filament spectrum reported in Figure 1, which shows ester carbonyl, C–O, and terephthalate ring absorptions and neither N–H stretching nor an amide II band. Accordingly, the present work characterises material–process systems as fabricated through commercially accessible additive manufacturing routes, rather than intrinsic bulk polymer properties; the implications of this choice are discussed at length in Section 4.

The materials selected for this study are supplied for biomedical or dental use, and the documentation supporting that designation, which varies in scope from material to material, is provided for each material in Table 1; it applies at the raw-material level. This certification applies to the material itself and does not extend to the final 3D-printed product, which would require separate validation to meet medical-device standards.

### 2.2. Specimen Fabrication

Before printing, each filament, for which a drying protocol is given in Table 2, was dried at the stated temperature and time to minimise moisture-induced extrusion defects, and kept in a dry cabinet until use.

Rectangular bar specimens (25 × 5 × 0.6 mm) were fabricated with geometry adapted from ISO 178 [27] and reduced to the thin cross-section relevant to contemporary orthodontics, which is dominated by aligner therapy. The 0.6 mm thickness was chosen for two reasons. It lies within the range of finished clear aligners, and it is the thickness that a 0.75 mm thermoformed sheet reaches after forming [28]. It also allows comparison with published specimen-level work: the same 0.6 mm section has been used in both a study of shape-memory aligner material [28] and our earlier testing of blended printable aligner resins [13], so the values reported here can be read against those datasets rather than in isolation. Specimens were printed on a 333 Dry (3D-Dream, Solbiate Arno, Italy) FDM printer with a 0.4 mm nozzle. All FDM specimens were printed flat, with the 25 × 5 mm face on the build plate, the long axis parallel to the X axis, and the 0.6 mm dimension along the build direction, so that deposition layers lay perpendicular to the loading axis in three-point bending. Common slicing settings were a 0.10 mm layer height (including the first layer), 0.40 mm line width, a classic wall generator with a single perimeter and no top or bottom shells, 100% rectilinear infill with automatic 0°/90° alternation between layers, a 20 mm/s first-layer speed, 1500–2000 mm/s^2^ acceleration, and the auxiliary fan disabled and ironing off; the seam was placed on a short end outside the central test region. Material-specific nozzle and bed temperatures, part-cooling fan, printing speed, volumetric flow, and filament drying are given in Table 2. PA 2201 (PA12 medical grade) was instead produced by the manufacturer (Materialise, Technologielaan Leuven, Belgium) by selective laser sintering on an EOS FORMIGA P 110 (30 W CO_2_ laser) (Robert-Stirling-Ring 1, Krailling, Germany) at a nominal 0.10 mm layer thickness; the service provider set the build orientation, the powder-refresh ratio, exposure settings and thermal cycle were not separately disclosed, and the supplier stated that the material is certified biocompatible following testing for cytotoxicity, sensitisation, irritation, acute systemic toxicity and pyrogenicity.

The PETG bed was set to 70 °C for the first layer and 65 °C thereafter, with the part-cooling fan at 20% from the third layer onward. PEEK was printed with a dedicated 0.4 mm nozzle at 395 °C (first layer at 400 °C) in a 90 °C-heated chamber (45–60 min preheat), with a 10 mm/s first-layer speed, a 5–8 mm brim, and slow cooling with the chamber closed. PA12 (PA 2201) was laser-sintered by Materialise on an EOS FORMIGA P 110 (30 W CO_2_ laser, nominal 0.10 mm layer); exposure and thermal parameters were set by the service provider and not disclosed. Drying denotes pre-print filament conditioning.

After printing, specimens were allowed to cool at room temperature and were visually inspected for warping or voids under a stereomicroscope. Dimensional accuracy was verified using a digital calliper (±0.01 mm), and defective samples were discarded. Five valid specimens per material (*n* = 5) were retained for testing.

### 2.3. Three-Point Bending Test

Mechanical behaviour was evaluated with a custom low-load three-point bending device built by the Department of Physics for orthodontic polymeric specimens, comprising two parallel cylindrical supports, a central loading nose, a calibrated low-capacity load cell and a motorised, displacement-controlled actuator; the device and its independent metrological validation have been described and peer-reviewed previously [29], and the present study applies that validated instrument to a new set of materials. The support span was 10 mm (5 mm from the centre to each support), which, with a 0.6 mm specimen thickness, gave a span-to-thickness ratio of 16.7. Before each session, the force chain was calibrated with traceable reference loads, and displacement was verified against a calibrated dial indicator. The load cell was zeroed and checked against a 100 g reference mass, and testing proceeded only when the reading matched 100 g. Loads were recorded in grams-force and converted to Newtons. Benchmarking against a calibrated universal testing machine showed excellent performance (100 g reference mass, 100.03 ± 0.18 g, and coefficient of variation 0.18%; Bland–Altman bias +0.03 g; intraclass correlation coefficient 0.999). Specimens were equilibrated for 24 h and tested under thermostatic conditions at 37 °C in a Fusayama–Meyer-type artificial saliva (NaCl 0.4, KCl 0.4, CaCl_2_·2H_2_O 0.795, NaH_2_PO_4_·2H_2_O 0.69, Na_2_S·9H_2_O 0.005 and urea 1.0 g/L; pH 6.8–7.0, adjusted with NaOH or HCl) [30].

A vertical load was applied centrally at a crosshead speed of 1 mm/min until 1 mm deflection, recorded in 20 equal increments (0.05 mm per step). Force–displacement data were continuously acquired and used to calculate:

- Apparent bending stress at 1 mm deflection (σ_1mm_);
- Flexural modulus (E_f_);
- Total energy (U_tot_);
- Dissipated energy (U_diss_).

From the loading–unloading curves, the unloading modulus was also calculated to assess elastic recovery.

Operationally, total energy (U_tot_) was computed as the integral of force over deflection during the loading branch up to 1 mm (U_tot_ = ∫F dx), and dissipated energy (U_diss_) as the area enclosed by the loading–unloading hysteresis loop in the cyclic test. The apparent flexural stress was σ = 3FL/(2bh^2^) and the mid-span strain ε = 6hδ/L^2^, with F as the recorded force, L as the span (10 mm), b as the width (5 mm), h as the thickness (0.6 mm) and δ as the deflection. The apparent flexural modulus was E_f_ = L^3^m/(4bh^3^), where m is the slope of the load–deflection curve over the 0–1 mm loading window, over which the response was near-linear (mean R^2^ = 0.995); the unloading modulus was obtained from the slope of the unloading branch over the same range. E_f_ and the flexural stress are reported throughout as apparent values. Two features of the configuration place it outside the assumptions of the small-deflection Euler–Bernoulli relations from which they are derived: the absolute specimen dimensions are far below the ISO 178 standard [27] geometry, and the imposed 1 mm deflection is 1.67 times the specimen thickness, so the test does not remain in the small-deflection regime. Transverse shear, by contrast, is a minor contributor at this span-to-thickness ratio, as quantified in the Discussion. The bending procedure was adapted from ISO 178 [27] to simulate intraoral conditions.

### 2.4. Stress-Relaxation Test

To assess time-dependent viscoelastic behaviour, stress-relaxation tests were performed on the same specimens under the same thermostatic conditions at 37 °C in artificial saliva, with the specimens maintained at a constant deflection of 1 mm for 10 min, and the applied stress was continuously recorded as it decayed. At each time point, the instantaneous flexural modulus E(t) was computed from the recorded force at the fixed 1 mm deflection using the same apparent-modulus expression applied in the bending test. The initial modulus (E_0_) was taken as E(t) at the onset of the hold (t = 0, as the first point recorded after the target deflection was reached) and the final modulus (E_final_) as the mean of the final three recorded moduli, which reduces the influence of instrumental noise. The percentage relaxation was defined as (E_0_ − E_final_)/E_0_ × 100. Because E_0_ is a secant value taken at the 1 mm hold onset in a separate relaxation test, whereas the flexural modulus reported in Table 3 is the slope over the 0–1 mm loading window of the bending test, the two quantities are defined differently and need not coincide; E_0_ is systematically the lower of the two, because the secant at 1 mm falls below the mean loading slope and a small amount of stress relaxation occurs in the brief interval between reaching the fixed deflection and recording the first hold point.

### 2.5. Statistical Analysis

The experimental unit was the individual specimen, and specimens were treated as independent. For each FDM material, the five specimens came from a single build, so they are replicates of a single manufacturing run rather than of the process; this is pseudoreplication at the build level, and the analysis therefore describes only within-build variability. The SLS PA12 specimens were manufactured externally and did not derive from a single dedicated build. The apparent flexural modulus was the primary mechanical outcome, and percentage stress relaxation was the primary time-dependent outcome. Apparent stress at 1 mm, the energies, and the unloading modulus are secondary and descriptive: under a fixed geometry, they are analytic transforms of the same recorded force, correlated with the primary outcome by construction, and not independent evidence.

Five specimens per material were tested, the number specified by ISO 178 [27]. No a priori power analysis was performed, and we would argue that it is of limited value here. Such a calculation rests on an assumed variance, which is informative when the variance is biological and largely outside the experimenter’s control. In a test on industrially produced specimens, the variance is a property of a controlled manufacturing process, the testing standard already prescribes the sample size, and the dispersion can be measured.

We therefore report the resolution that the design actually achieved rather than a pre-study estimate of it. With the measured within-material coefficient of variation of 6.2%, five specimens per group resolve a difference of about 13% of the mean in a single pairwise comparison at 80% power and α = 0.05, rising to about 20% once α is corrected for the fifteen pairwise comparisons. The analysis actually reported is more conservative than that calculation because it uses rank-based Dunn tests with Holm correction across fifteen pairs rather than a single two-sample comparison, and rank tests on five observations per group carry little power. What the design achieved is therefore what Section 3.1 reports: only the extremes of the distribution separated, while adjacently ranked materials did not, including pairs whose means differ by a quarter or more. The design supports the order-of-magnitude comparison the study was built to make and does not support inference about adjacent rankings. Because a single-build design cannot estimate between-build variability, a second independent build of PETG and PC was printed after the main experiment, using the same spools, drying protocol, slicing parameters, and printer, with three specimens of each material tested identically. As with the quantitative FTIR comparison, this was added at revision and is post hoc with respect to the original design; three specimens from one further build cannot estimate a between-build variance and offer only a single independent point of comparison for two of the six systems.

Descriptive statistics are reported as mean, standard deviation, and t-based 95% confidence intervals (*n* = 5); because inference was rank-based, medians and interquartile ranges are additionally tabulated in the Supplementary File S1, while means with confidence intervals are given in the main text for interpretability. The distribution of each endpoint was screened with the Shapiro–Wilk test, whose power is limited at this sample size, so non-parametric methods were adopted as a precaution. Between-material differences were assessed with the Kruskal–Wallis test, followed by Dunn post hoc pairwise comparisons with Holm correction for multiple testing, with a two-sided *p* < 0.05 taken as significant; epsilon-squared (ε^2^) is reported as the Kruskal–Wallis effect size. For percentage relaxation, TPC was excluded from the inferential analysis because, at its very low forces, the modulus change over the hold approached the load-cell resolution and could not be reliably quantified. Analyses were run in Python 3.11 (SciPy 1.11 and scikit-posthocs 0.8).

No equivalence testing was performed, and no equivalence margin was defined. A comparison that did not reach significance therefore indicates only that this sample was unable to resolve a difference; it must not be read as evidence that two materials behave alike. Every between-material statement other than those explicitly reported as significant is descriptive.

### 2.6. FTIR Spectroscopy

Fourier-transform infrared (FTIR) spectroscopy was used as a process-validation step for the FDM-processed materials (PETG, PC, PA, PEEK, and TPC), in which melt extrusion, shear-induced thermal history, and layer-by-layer reheating create opportunities for chemical alteration. For each material, the spectrum of the virgin filament and that of the printed specimen were acquired under identical conditions and overlaid, so that any processing-induced change in functional-group chemistry would appear as a new band or as the loss or shift in a characteristic band. Spectra were recorded in attenuated total reflectance (ATR) mode with a diamond ATR module across 4000–600 cm^−1^ at 4 cm^−1^ resolution, averaging 32 scans per measurement, with a background acquired between samples. Each material was measured in triplicate; spectra were smoothed with a Savitzky–Golay filter, normalised to the maximum absorbance peak and averaged for comparison. Agreement between the paired virgin and printed spectra was then quantified rather than judged visually: each spectrum was normalised to its own maximum absorbance, and the Pearson correlation coefficient between the two was computed point-by-point over the full 7052-point range (600.7–4000.1 cm^−1^). Because a correlation coefficient is interpretable only relative to a reference, the same coefficient was also computed between spectra of different materials (the discriminant control), and a threshold of r > 0.95 was adopted as the criterion for spectral agreement, fixed by reference to that control before the paired coefficients were examined. The comparison was added at revision and is therefore post hoc with respect to the original study design. FTIR here validates the FDM extrusion route, in which melt processing could alter the polymer, rather than serving as a symmetric process-comparison endpoint. PA 2201 is a certified medical-grade laser-sintering powder used as supplied, and the study compares material–process systems as realistically produced rather than a single polymer across two processes. The SLS-processed PA12 could not be included in this comparison for practical reasons: the specimens were manufactured externally by the service provider, so the virgin powder was never in our possession, and no paired virgin-versus-printed measurements were possible. The comparison would be worth making, since localised laser heating could, in principle, produce surface oxidation or a change in crystallinity, and it would require access to the unprocessed powder.

## 3. Results

### 3.1. Three-Point Bending Behaviour

**Figure 2:**
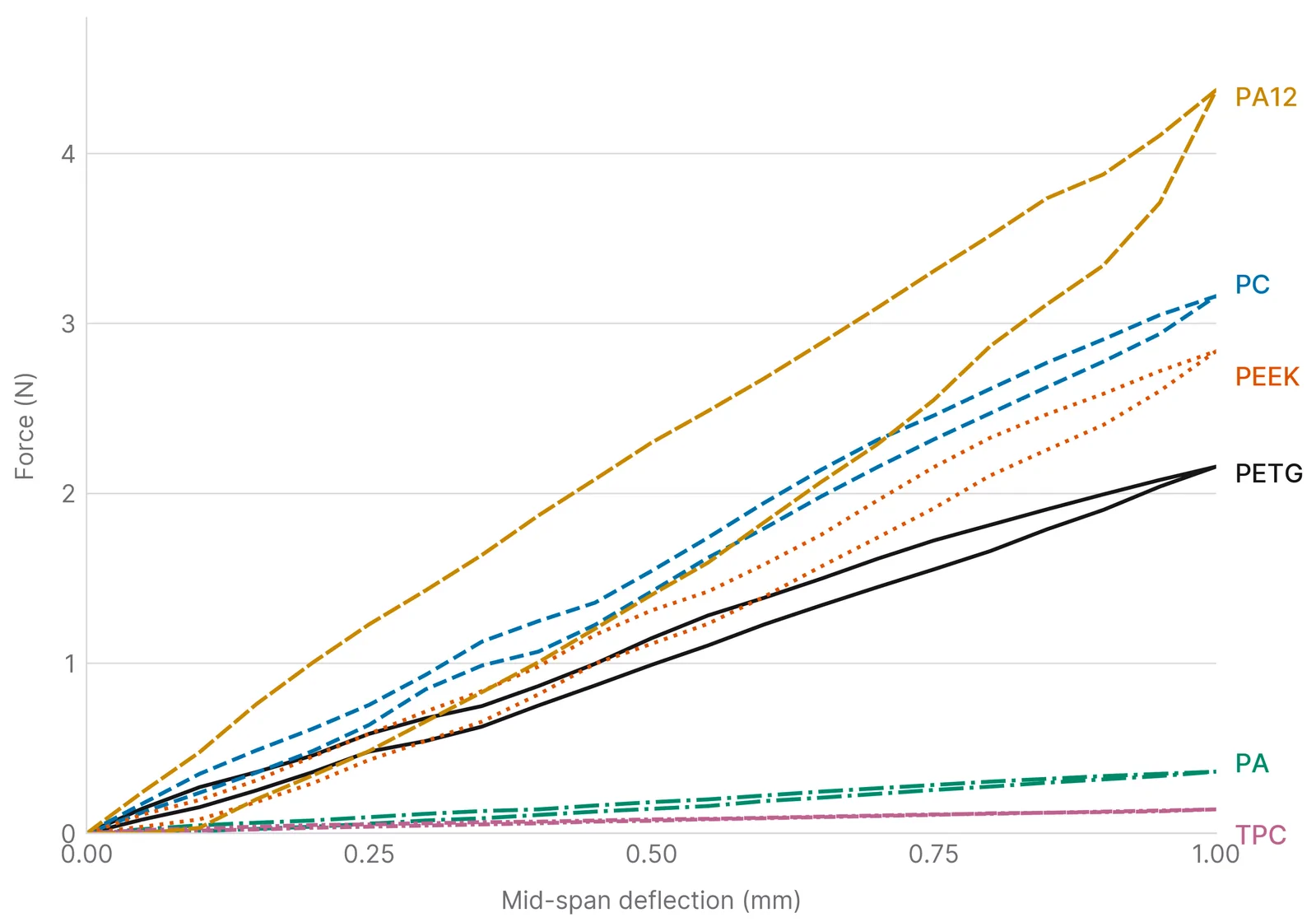
Three-point bending curves of all tested materials.

The six thermoplastic materials showed distinct load–deflection behaviours under three-point bending (Figure 2); mean parameters with 95% confidence intervals are given in Table 3 (*n* = 5).

PA12, PC and PEEK produced the steepest loading branches and the highest apparent flexural stiffness; PETG was intermediate, and PA and TPC were markedly more compliant. Hysteresis between loading and unloading was limited for the stiffer polymers, consistent with a predominantly elastic response, and largest in absolute terms for PA12. For TPC, the very low forces generated at 1 mm deflection produced minimal hysteresis, reflecting the low-strain regime rather than an intrinsic inability to dissipate energy.

The material–process system had a very large effect on apparent flexural stiffness (Kruskal–Wallis H = 27.6, *p* < 0.001; ε^2^ = 0.95). As set out in Section 2.5, these tests treat the five specimens of each material as independent, so every *p*-value below involving an FDM material is conditional on the single build from which its specimens came and describes within-build rather than process-level variation. The independent verification build addresses how much this matters in practice: it gave mean apparent flexural moduli (*n* = 3) of 513 MPa for PETG and 743 MPa for PC, deviating by +0.7% and −0.9% from the means of the original builds and falling well inside their 95% confidence intervals (469.9–548.8 and 692.4–807.7 MPa). For scale, the within-build coefficient of variation, computed from the standard deviations reported in the Supplementary File S1 rather than from the confidence intervals of Table 3, was 6.2% for both materials, and the ±0.01 mm calliper tolerance alone propagates to about 5% on the modulus, since the modulus scales with the cube of thickness. The two build means therefore differed by less than the dispersion among specimens of a single build, although the two quantities are not directly comparable: one is a difference between two means, and the other a dispersion among five specimens. Dunn comparisons with Holm correction resolved only the extremes of the distribution. TPC differed significantly from PC, PEEK and PA12, and PA differed from PA12 (*p* < 0.05). Adjacently ranked materials, including the PETG–PC, PEEK–PETG and PC–PA12 pairs, did not reach significance at this sample size. Those comparisons are therefore exploratory: the *p*-values are read descriptively, and the effect sizes, though large, are imprecisely estimated. Where significance was not reached, the numerical ordering of adjacent materials is given for completeness only and is not an established ranking. Apparent flexural stress at 1 mm is a linear transform of the same force at 1 mm deflection and therefore returns an identical ranking; it, and the energy descriptors that are analytic functions of modulus and stress, are reported for completeness rather than as independent evidence.

Table 3 summarises the corresponding parameters. The stiffness hierarchy is mirrored by stress at 1 mm, stored and dissipated energy, and unloading modulus, with PA12 as the highest and TPC as the lowest throughout; for PA, the unloading and loading moduli coincide within their confidence intervals. Expressed as a fraction of the work of loading, the dissipated energy was also largest for PA12 (U_diss_/U_tot_ about 0.38, against about 0.17 for PC and 0.19 for PETG), so PA12 is the most dissipative material in relative as well as absolute terms; TPC dissipated energy fell at the measurement floor and rounds to zero.

### 3.2. Stress-Relaxation Behaviour

**Figure 3:**
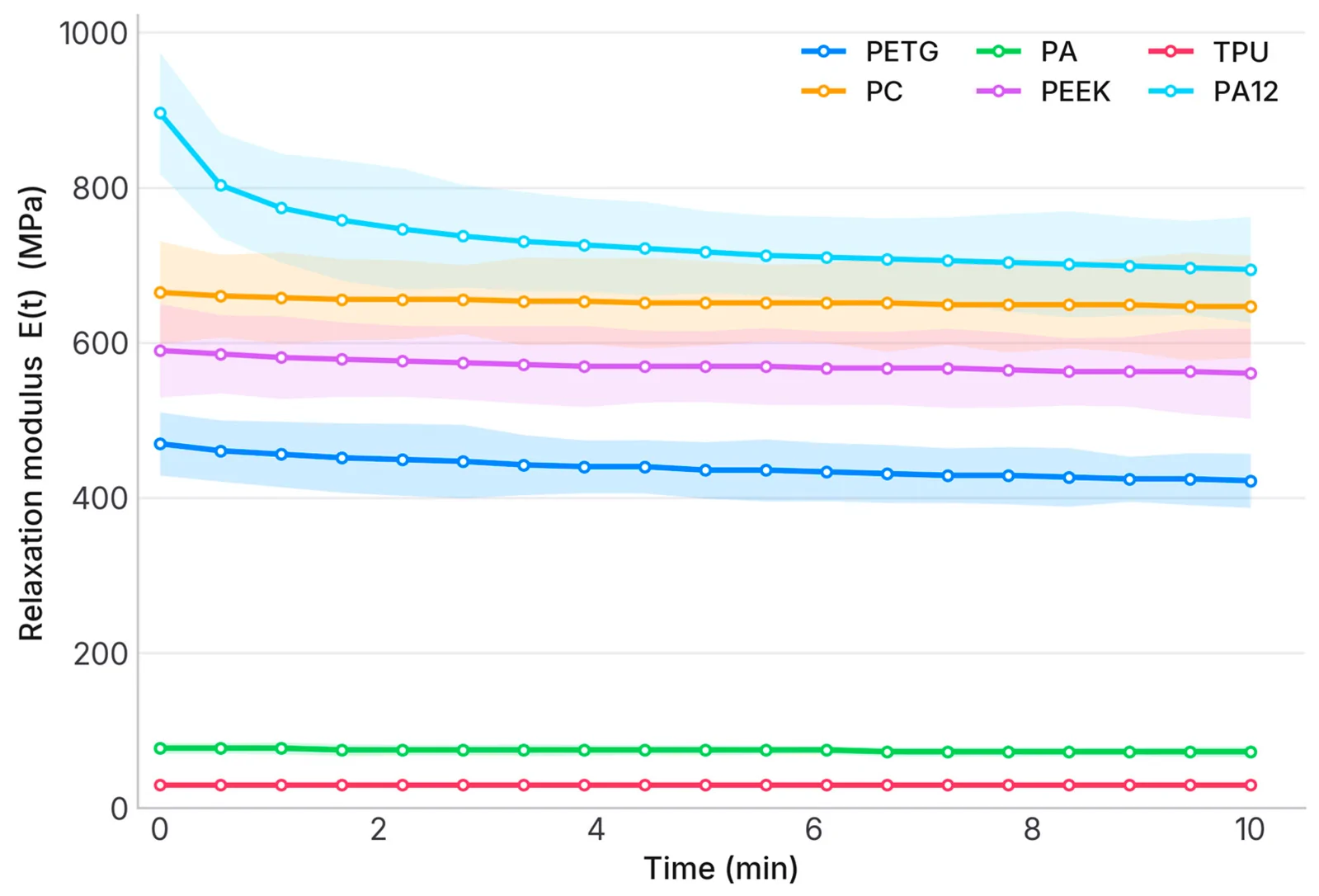
Stress-relaxation behaviour: mean relaxation modulus E(t) with 95% confidence bands over 10 min at a constant 1 mm deflection. The Shadow is the standard deviation.

Figure 3 shows the mean relaxation modulus E(t) with 95% confidence bands over 10 min at a constant 1 mm deflection. All materials retained most of their initial modulus. The stiff polymers decayed the least; PA12 showed the greatest decay despite having the highest absolute modulus, and for TPC, the low forces resulted in a modulus change below reliable quantification.

**Figure 4:**
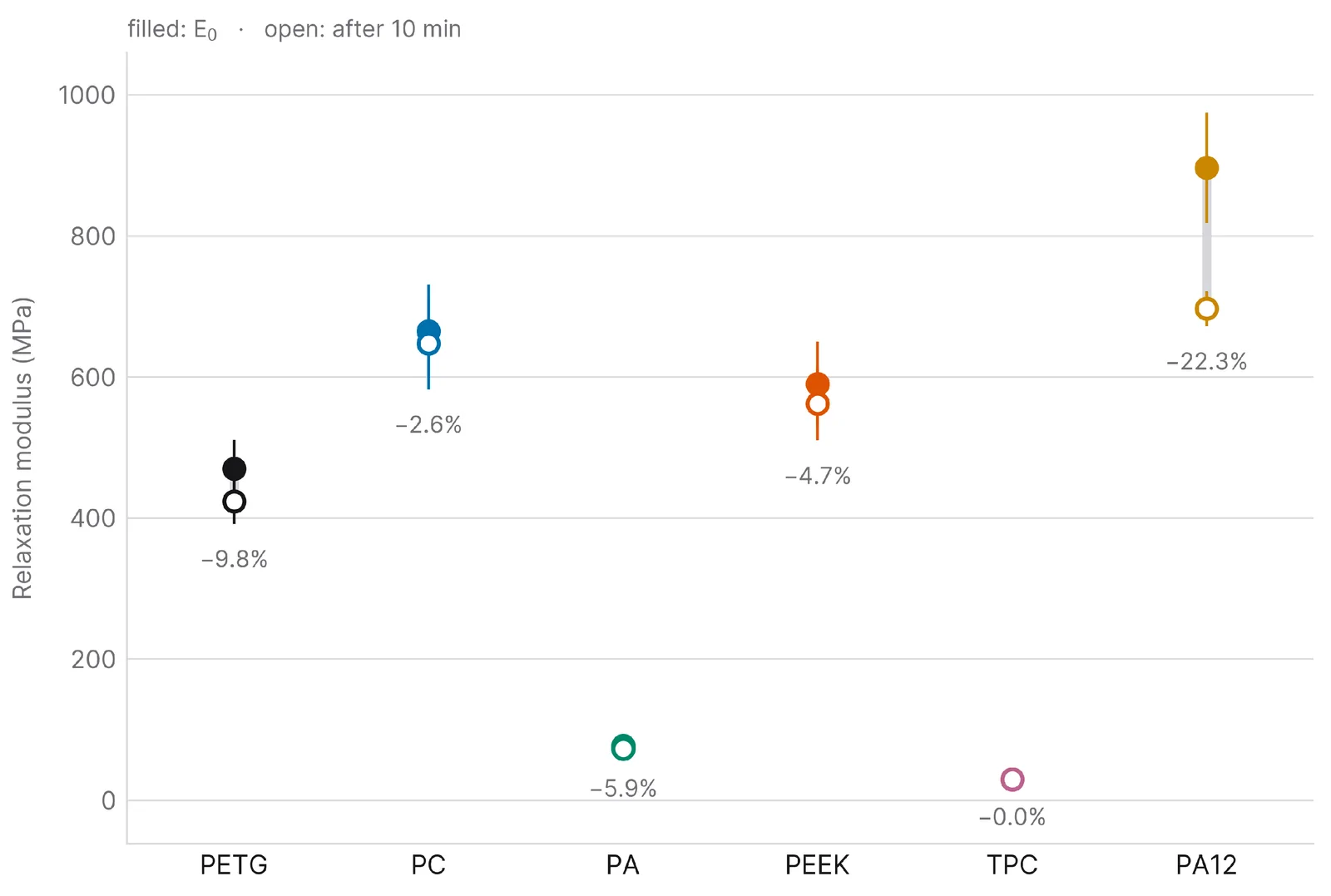
Evolution of the stress-relaxation modulus: initial (E_0_) versus final (E_final_) modulus for each material (mean, 95% CI, *n* = 5). The filled dot is the initial value, the empty dot is the final value. The bars are the standard deviations.

Figure 4 compares the initial modulus (E_0_) with the final one (E_final_, the mean of the last three recorded moduli), as means with 95% confidence intervals (*n* = 5). E_0_ and E_final_ were 470 ± 41 and 424 ± 32 MPa (PETG), 665 ± 66 and 648 ± 65 (PC), 77 ± 7 and 73 ± 6 (PA), 590 ± 60 and 562 ± 52 (PEEK), 30 ± 3 and 30 ± 2 (TPC), and 897 ± 78 and 697 ± 25 (PA12). Only PA12 showed a marked reduction; the initial and final moduli of the other materials overlapped within their confidence intervals.

Because a percentage is hard to interpret clinically, the same data are also expressed as absolute force at the fixed 1 mm deflection (Table 4); under the present geometry, the two are related by F [N] = 0.00432 × E [MPa]. Read this way, the ranking changes meaning: PA12 lost by far the most force in absolute terms (0.86 N), yet the 3.01 N it still delivered after 10 min was higher than the initial forces of PEEK, PETG, PA, and TPC. It was not separable from the initial force of PC, whose confidence interval (2.59–3.16 N) contains it. In absolute terms, the smallest loss was PA’s (0.02 N), which reflects how little force it generated; among the materials delivering more than 1 N, PC lost the least (0.08 N) and retained 97.4%.

**Figure 5:**
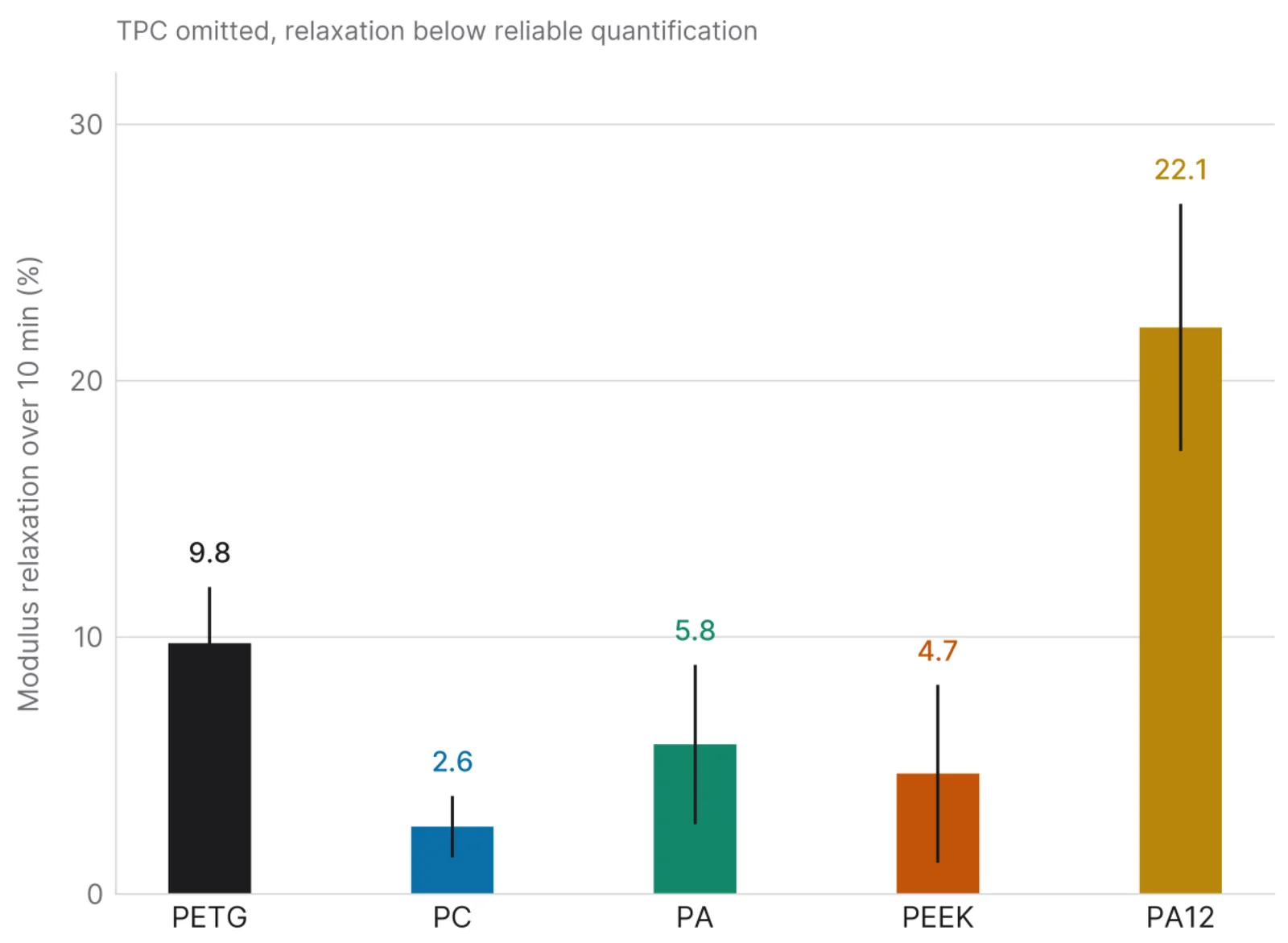
Modulus relaxation percentage over 10 min (mean, 95% CI, *n* = 5). TPC is not shown because its relaxation fell below the limit of reliable quantification.

Geometry-specific comparative estimates for a 25 × 5 × 0.6 mm bar at 1 mm mid-span deflection, not forces delivered by an appliance to a tooth. Each column is rounded independently from the unrounded values, so differences and ratios recomputed from the rounded figures shown may differ in the last digit. Force retained is computed as 100 − mean percentage relaxation (Figure 5), that is, as the mean of the per-specimen ratios rather than the ratio of the mean forces. n.q., not quantifiable: for TPC, the change over the hold did not exceed the resolution of the load cell, so no decay figure is reported.

Figure 5 reports the percentage modulus relaxation over 10 min (mean, 95% CI, *n* = 5). For TPC, the modulus change over the hold approached the load-cell resolution (relaxation −0.1 ± 2.7%, indistinguishable from zero), so its percentage relaxation could not be reliably quantified, and TPC was excluded from this analysis. Among the five measurable materials, the effect was significant (Kruskal–Wallis H = 19.2, *p* < 0.001; ε^2^ = 0.80). PA12 showed the greatest relaxation (22.1 ± 4.8%) and differed significantly from PC (2.6 ± 1.2%) and PEEK (4.7 ± 3.5%) in Dunn comparisons with Holm correction; the remaining pairs, including PETG (9.8 ± 2.2%) and PA (5.8 ± 3.1%), did not reach significance, so these comparisons are exploratory.

### 3.3. FTIR Spectra

Every FDM-processed thermoplastic showed its expected chemical signature (Figure 1): the ester carbonyl and aromatic absorptions of PETG, the carbonate and aromatic bands of PC, the amide I and II bands of PA, the aromatic C–C stretching peaks of PEEK, and the ester carbonyl (1713 cm^−1^), C–O (1269 and 1101 cm^−1^) and terephthalate ring (727 cm^−1^) absorptions of the TPC copolyester elastomer.

For every material, the virgin-filament and printed-specimen spectra were overlaid closely, with Pearson correlation coefficients of 0.9995 (TPC), 0.9978 (PETG), 0.9923 (PA), 0.9883 (PC), and 0.9869 (PEEK). All exceeded the 0.95 threshold. Computed between spectra of different materials, the same coefficient ranged from −0.30 to 0.80, so the agreement observed within each virgin–printed pair is clearly separated from the discriminant control. No new absorption band appeared above the baseline noise, no characteristic band was lost, and no band shift was apparent.

Residual differences were small but not uniform across materials. They were the smallest for TPC and PETG (root-mean-square differences of 0.005 and 0.011 in normalised absorbance) and the largest for PEEK, PC and PA (0.041, 0.033 and 0.030). For PC and PEEK, they lie mainly above 3100 cm^−1^, a region to which atmospheric water vapour and baseline drift commonly contribute in attenuated total reflectance. For PA, the largest single difference (0.18 in normalised absorbance) fell at 1711 cm^−1^, within the carbonyl region.

## 4. Discussion

### 4.1. Stiffness and Force Retention Across the Six Systems

Three-point bending and stress relaxation characterised six thermoplastic material–process systems supplied for biomedical or dental use. The PA12/SLS system, PC and PEEK were the stiffest (though adjacent ranks were not statistically separable at *n* = 5; no failure strength was measured, since loading stopped at a fixed deflection), with apparent flexural moduli of roughly 989, 750 and 687 MPa and flexural stress at 1 mm above 23 MPa; PA12 also showed the highest absolute hysteresis. The unloading modulus closely matched the loading modulus, indicating a predominantly elastic response. Under sustained 1 mm deflection, PC and PEEK relaxed only slightly (2.6% and 4.7%), whereas PA12, despite its higher stiffness, relaxed by 22.1% and showed significantly greater relaxation than PC and PEEK; this greater relative decay may reflect the combined influence of the PA12 molecular architecture and its SLS-related thermal and microstructural history, and is relevant when sustained-force stability is required. PEEK stiffness in particular is sensitive to crystallinity, and the 90 °C chamber and slow closed-door cooling used here favour crystallisation, so the reported PEEK values reflect that specific thermal history.

PETG occupied an intermediate position, with an apparent flexural modulus of about 509 MPa and a flexural stress at 1 mm of roughly 18 MPa. It showed intermediate absolute energy dissipation, and its modulus relaxed progressively over the 10 min window (mean 9.8%), consistent with a mild viscoelastic response that places it between the rigid engineering polymers and the more compliant polyamides.

PA exhibited a markedly lower apparent flexural modulus (approximately 85 MPa) and, correspondingly, lower energy absorption at 1 mm. Its mean relaxation was moderate (5.8%), so it retained most of its initial modulus after 10 min, although at *n* = 5, this value was not statistically separable from those of the other intermediate and compliant materials. Its low absolute stiffness, well below that of typical bulk polyamide, probably reflects moisture uptake in this hygroscopic filament together with interlayer porosity from thin-section FDM, rather than an intrinsic property of the polymer alone.

TPC was the most compliant polymer, with an apparent flexural modulus of about 32 MPa. The forces it generated at 1 mm deflection placed its stress relaxation below the reliable resolution of the setup, so its percentage relaxation could not be quantified, and this should not be read as an absence of viscoelasticity; copolyester elastomers of this class are intrinsically viscoelastic and dissipate substantial energy at larger deformations through their soft segments. Its low apparent stiffness and the low forces recorded here were measured at a single thickness (0.6 mm) and in a single printing configuration. We therefore cannot attribute them to the geometry rather than to the material: that reading is consistent with the elastomeric nature of TPC and with reports that appliance thickness governs the force delivered [31,32], but it was not tested here, because no other thickness was printed. What the data establish is narrower: at 0.6 mm, TPC delivers forces close to the setup’s lower sensitivity limit. Whether greater wall thickness would bring it into a useful range is a prediction that would require a thickness series to confirm. TPC was retained in the flexural comparison and excluded only from the relaxation inference. The distinction is between a force and a change in force: the 0.13 N it generated at 1 mm is small but measurable, whereas the change in that force over the 10 min hold was not distinguishable from the resolution of the load cell.

### 4.2. Short-Term Relaxation and the Choice of a 10 min Window

The relaxation values observed for the FDM and SLS thermoplastics (ranging from 2.6% to 22.1% over 10 min among the measurable materials) are lower than those reported for conventional thermoformed aligner materials. Lombardo et al. reported that thermoformed polyurethane and PETG aligners lost more than 50% of their initial load over 24 h of sustained deflection, with material-specific differences in the magnitude and rate of decay [33]. The comparison is qualitative, given the differences in test duration, deflection mode, and specimen geometry; within the present dataset, PC and PEEK relaxed the least, PETG was intermediate, and PA12 retained the highest absolute modulus despite the greatest relative relaxation.

The 10 min window was chosen because the curves had substantially flattened by that point, and the recorded data bear this out. Between 69% and 89% of the total decay had already occurred within the first five minutes, depending on the material, and over the final two minutes, the rate of decay had fallen to between 6% and 33% of its initial value, the smallest ratio being that of the PA12/SLS system, which decayed fastest at the outset. Extending the hold would therefore have added time without adding discrimination between materials, which is what a screening comparison requires. It measures a different quantity from appliance durability, and the distinction is as much one of object as of timescale. Here, the object is a bar of material, and the question is how its stiffness compares with that of five others; there, the object is a finished appliance, and the question is how long it lasts in the mouth. The second question is answered by testing the appliance over the weeks or months it is meant to serve, not by holding a specimen longer on a bench. The comparison with the >50% loss reported over 24 h for whole thermoformed aligners [33] is presented as qualitative for that reason.

### 4.3. Why the Apparent Moduli Are Low

The apparent flexural moduli are well below published bulk values for these engineering polymers, as expected from the short span (10 mm) and thin cross-section (0.6 mm). Although the L/h ratio of 16.7 is close to the ISO 178 nominal value of 16, the absolute dimensions are far below the standard 80 × 10 × 4 mm geometry. Hence, the configuration is a deliberate adaptation rather than a standard-compliant test [27]. Two distinct effects follow, and it is worth separating them because they are easily conflated.

The first, transverse shear, is small. In Timoshenko-beam terms, the ratio of shear to bending deflection under central loading is fs(E/G)(h/L)2, which, for a rectangular section, reduces to 2.4(1 + ν)(h/L)^2^. With h/L = 0.06 and Poisson’s ratios spanning 0.30–0.48, which covers the polymers tested, shear accounts for 1.11–1.26% of the total measured deflection (the shear-to-bending ratio itself spans 1.12–1.28%). Correcting each apparent modulus by its own factor raises PA12 from 989 to 1001 MPa, PC from 750 to 759 MPa and TPC from 32.3 to 32.7 MPa, and leaves the ordering unchanged: the correction varies by only 0.15 percentage points across the six materials. Shear, therefore, neither explains the gap in bulk values nor alters the relative ranking. All moduli quoted elsewhere in this paper are the uncorrected apparent values; the shear-corrected figures are given here only to show the size of the correction.

The second effect is larger. The imposed 1 mm deflection is 1.67 times the specimen thickness, so the test lies outside the small-deflection regime in which the Euler–Bernoulli relations applied here are valid. Together with the printed thin section (a single perimeter, no top or bottom shells, and interlayer boundaries occupying an appreciable fraction of a 0.6 mm thickness), this is the more plausible origin of the low absolute values. The first of these is purely geometric and acts identically on all six materials. The second is not: the printed architecture differs between the five extruded materials and the laser-sintered PA12, which has no perimeter-and-infill structure, so this contribution is not common to the set and is one more reason to read the PA12 values as belonging to a material–process system. The relative hierarchy nonetheless holds across a range far wider than any of these effects, with PEEK about eightfold and PA12 about twelvefold stiffer than PA, and both more than twentyfold stiffer than TPC. Because this thin-section geometry mirrors the cross-section of printed aligners and auxiliaries, the apparent values are more informative for comparing candidate materials than bulk figures would be. They remain at the specimen level and do not describe how a finished appliance behaves in service.

### 4.4. Clinical Force Ranges and the Tooth Movements These Systems Could Serve

In this thin-section geometry, all materials showed apparent flexural moduli below or near 1 GPa. PA12 (about 989 MPa) approaches the lower boundary of conventional thermoformed aligner materials (1–2 GPa) [14], with PC (about 750 MPa) and PEEK (about 687 MPa) lower. The present specimen-level measurements cannot directly predict the forces generated by a finished orthodontic appliance, whose shell geometry, undercuts, fit and thickness distribution alter the force system. For orientation, the recommendations of Proffit as summarised by Sharif et al. [11] place useful orthodontic forces at 0.1–0.2 N for intrusion, 0.7–1.2 N for tipping and bodily movement, and 1.0–1.5 N for rotation control, and the same study measured three-point bending forces of 0.3–2.7 N on printed aligner materials. The forces recorded here at 1 mm deflection (Table 4) span 0.13–3.87 N and therefore cross that range rather than sitting inside it. TPC’s 0.13 N falls within the intrusion band; PA’s 0.33 N lies between the intrusion and tipping bands; PETG, PEEK, PC, and PA12, at 2.03–3.87 N, exceed the upper end of all three bands. This is a statement about one deflection, not about the polymers: force scales with the cube of thickness and linearly with activation, so the comparison depends on the 0.6 mm section and the 1 mm deflection used here. This is the reason the mapping below is framed as a hypothesis rather than a selection rule.

On that basis, the six systems are sorted into candidate application classes. Genuinely translucent PETG and PC, which retained 97.4% of their force over the hold, are the candidates for aligner-type applications, based on stiffness and force retention rather than on equivalence to thermoformed materials, whose 1–2 GPa none of the systems tested reach. The PA12/SLS system and PEEK, the stiffest and both opaque, may warrant device-level testing for rigid single-material components such as palatal bars and expansion auxiliaries, where stiffness and dimensional stability govern and appearance does not. TPC and PA, the most compliant, may warrant it for components designed around large elastic deformation; TPC in particular falls within the stiffness range of the elastomers used for prefabricated myofunctional appliances rather than that of tooth-moving devices. This mapping is a hypothesis generated by specimen-level data, not a clinical recommendation: no assignment was tested at appliance level, and each would require device-level mechanical, dimensional, and biological evaluation first.

The response is nearly linear over the tested window (mean R^2^ = 0.995), so the force a system delivers scales with the applied activation. The question of which movements it could serve can therefore be put in concrete terms: how far must a 0.6 mm section be deflected to reach the force each movement requires? Table 5 answers the three bands quoted above.

Force bands from Proffit as summarised in [11]. Deflections are those of a 25 × 5 × 0.6 mm bar on a 10 mm span, not of an appliance on a tooth. Values above 1 mm fall outside the tested window and are linear extrapolations. Entries above about 2 mm are given for completeness: a deflection of a fifth of the span or more is not a regime this geometry, or the linear relation used, can be taken to describe.

Read this way, the six systems separate into two groups. PA12, PC, PEEK and PETG reach all three bands within sub-millimetre activations, between 0.02 and 0.68 mm, which is the regime in which an aligner or an auxiliary actually works. PA and TPC reach only the intrusion band. PA covers it entirely, between 0.27 and 0.54 mm, while TPC reaches its lower end at 0.72 mm and would need 1.45 mm for the upper end, beyond the 1 mm tested. Neither comes near the tipping or rotation bands at this thickness, which would require between 1.9 and 10.8 mm of mid-span deflection on a 10 mm span.

For TPC, this is arguably the wrong yardstick. Its compliance, 32 MPa, with a force decay too small to quantify relative to the load cell’s resolution, places it among the elastomeric materials used for prefabricated myofunctional appliances such as trainers, rather than with materials that deliver a defined force to an individual tooth. Appliances of that class work through arch-level guidance and interaction with the perioral musculature, and their mechanical requirement is large, repeatable elastic deformation rather than a force held within the bands quoted above. Measured against those bands, TPC appears inadequate; measured against the appliance class it actually resembles, it may be appropriate. Which of the two readings holds is a question for device-level testing, and we make no claim on it here.

A more direct comparison is available. Sharif et al. [11] measured force on complete printed aligners at a 0.4 mm deflection and reported 0.3–0.8 N for extrusion, 1.2–2.7 N for intrusion and 0.4–1.7 N for oro-vestibular translation. These are forces measured on whole appliances in a bench test, not forces recommended for delivery to a tooth, which is why they lie an order of magnitude above the Proffit bands used in Table 5 for the same movements. At that same deflection, the systems tested here deliver 1.71 N (PA12), 1.30 N (PC), 1.19 N (PEEK), 0.88 N (PETG), 0.15 N (PA), and 0.06 N (TPC). Four of the six, therefore, fall within the ranges measured on real printed aligners: PA12 and PC in the intrusion range, and PEEK and PETG in the range for oro-vestibular translation. In contrast, PA (0.15 N) and TPC (0.06 N) fall two- and fivefold below the lowest of those ranges.

This is the sense in which the present measurements bear on tooth movement, and it is a bounded one: a rectangular bar in three-point bending is not an appliance, its deflection is not a programmed tooth displacement, and no moment, attachment geometry, or fit is represented. What the comparison supports is the narrower statement that four of the six systems generate force of the right order at the right activation, which is a precondition for their use and not a demonstration of it.

### 4.5. Comparison with Photopolymer Resins, and the Appliance Intended

Comparative studies of 3D-printed orthodontic aligners have reported that the mechanical properties of the printed resins depend strongly on the printing technology used. Aligners produced by LCD printers display higher hardness and indentation modulus than those produced by DLP printers [12]. This reflects a fundamental difference in test methodology: indentation modulus captures the local surface stiffness of a photocured resin layer, whereas the apparent flexural moduli reported here are governed by the thin-beam geometry (L/h = 16.7) of our specimens. Both datasets remain clinically meaningful within the constraints of their respective test designs, and a direct numerical comparison between the two methods is inappropriate [12,27]. It can be concluded that FDM thermoplastics and photocured dental resins occupy different regions of mechanical behaviour: the former span a broad range of apparent stiffness governed by bulk polymer properties, while the latter exhibit a narrower, surface-hardness-dominated range that is sensitive to printer type and post-curing conditions.

The six systems span a broad range of mechanical behaviour, from stiff (PA12, PC, and PEEK) to compliant (PA, TPC). Stiffness and force stability do not travel together: PC and PEEK combined high stiffness with little decay, whereas the PA12/SLS system was the stiffest but relaxed the most and dissipated the largest fraction of the loading work. This variability is what makes material selection meaningful for the force–deflection response of an appliance, although translating it into a design is beyond what specimen-level data can support. Whether stiff polymers would require reduced thickness or a hybrid construction to bring force levels into a useful range is a design question that this study does not answer. Materials of intermediate stiffness, such as PETG and PA, sit between the two extremes. At the same time, TPC generated the lowest forces in the set at the single thickness tested and would be a candidate only for components designed around large elastic deformation. Wall thickness is, in any case, a primary determinant of the force a printed appliance delivers [31,32].

A broader consideration concerns the intended appliance. Additive manufacturing in orthodontics is not limited to clear aligners: single-material components such as palatal bars and arches, retainers, and skeletal or expansion auxiliaries are increasingly printed, and for these the governing requirements are stiffness, dimensional stability and biocompatibility rather than optical clarity. Of those three, only stiffness was characterised here; dimensional stability and biocompatibility were not measured. Among the materials tested, PETG is genuinely translucent, PA (Nylon 680) is semi-translucent, PC is translucent with a blue tint, and PEEK, PA12 and TPC are opaque; this restricts the opaque materials for full-time aesthetic aligners but not for the many appliances in which appearance is secondary, and opaque or part-time appliances are already in routine clinical use. Transparency, haze, and surface finish were not evaluated in the present work and would require dedicated optical and dimensional testing before any material is proposed for aesthetic aligners. It should also be noted that commercial full-time clear aligners are frequently multilayer, combining a stiffer layer with a more compliant one, an architecture that the single-material specimens tested here do not reproduce; the present data therefore inform material and process selection for orthodontic appliances and auxiliaries in general rather than the validation of a finished clear aligner.

### 4.6. Chemical Stability of the Printed Material

The FTIR spectra complement the mechanical results. Quantifying the paired comparison, rather than describing the spectra as superimposable, places this evidence on a firmer footing: correlation coefficients of 0.987 or higher, compared with a discriminant control that does not exceed 0.80 between different materials, indicate that FDM processing produced no detectable change in the functional-group profile under the conditions adopted.

The residual difference observed for PA warrants comment, as it is the largest in the set and occurs at 1711 cm^−1^, within the carbonyl region. Extrusion at 255 °C could, in principle, initiate oxidation, and an increase in carbonyl absorption would be the expected signature. A difference of this magnitude is equally compatible with baseline drift between two uncorrected spectra. The present data, one averaged spectrum per state and uncorrected for baseline, cannot distinguish the two, and a dedicated investigation with baseline correction and replicate acquisitions would be needed. We report it rather than resolve it. FTIR is, in any case, insensitive to changes such as molecular-weight or crystallinity variation, and was not acquired on the SLS-processed PA12.

The FDM route is also chemically simpler than photopolymer printing, which motivates this study’s biocompatibility framing without demonstrating it. Photopolymer resins are formulated with photoinitiators, reactive diluents, and cross-linkers that are associated with cytotoxicity in dental and periodontal cell models [16]; direct-printed aligners release more microplastic and nanoplastic particles under simulated oral conditions than thermoformed devices [19]; and urethane-based photopolymer particles can shift macrophages towards pro-inflammatory phenotypes [20]. FDM filaments, by contrast, are pre-polymerised single-component thermoplastics, and the paired FTIR comparison showed no gross change in functional-group profile after processing for the FDM materials. The relevance of this to the biological question is narrow but real, and it is worth being exact about the division of labour. Establishing the biological safety of a polymer is the manufacturer’s task, carried out in accordance with ISO 10993 [21] and comparable standards for the raw material, and the resulting documentation is summarised by material in Table 1. Because these polymers are supplied fully polymerised, that documentation is inherited by the printed part only insofar as printing leaves the polymer chemically unchanged. Testing that premise is something a study of this kind can do, and it is why the paired comparison was made: the spectroscopic agreement reported above is evidence on precisely that point. It is not evidence of biocompatibility: qualitative ATR-FTIR cannot exclude oligomer, additive, or particulate release from the printed geometry, the certification applies to the material and not to a finished device, and FTIR was not acquired on the SLS-processed PA12 because its specimens were manufactured externally and the virgin powder was not available to us. Cytotoxicity, particulate release, surface quality and intraoral ageing of these specimens were not assessed and remain necessary before any clinical recommendation; the present data show only that PC combines useful stiffness with the highest force retention measured here (97.4%), and that PETG offers a lower but still moderate retention (90.2%) at an intermediate stiffness. Their apparent moduli remain below the 1–2 GPa reported for thermoformed aligner materials, and the comparison of relaxation with those materials is qualitative, so no claim of mechanical equivalence to them is made.

### 4.7. Limitations

Several limitations bound what these data support, and most are consequences of the design stated in Section 2.5 rather than additions to it.

The design bounds the inference. Five specimens per material, from a single build, were treated as independent; the comparisons resolve only the extremes, and every other between-material statement is exploratory. The verification build provides one independent comparison on three specimens for two of the five FDM materials; it does not extend to PA, PEEK, TPC, or the externally manufactured PA12, and a further build indicates the likely scale of between-build variation without estimating its variance. Because PA12 was the only laser-sintered material, polymer and process effects cannot be separated for it, and the five FDM values are likewise specific to the extrusion route used; partitioning the two would require FDM-printed PA12 under the same protocol.

Specimens were rectangular bars, not appliance geometries, and the reported moduli and stresses are apparent values, internally comparable but not bulk properties. Thickness was held within ±0.01 mm, which corresponds to roughly 5% in modulus because modulus scales with the cube of thickness; this contribution is not separated from material variation in the reported intervals. All FDM specimens were printed in one orientation. The 10 min relaxation window characterises the initial decay of the material and not the day-to-week behaviour of an appliance, which is a separate measurement on a separate object; the comparison with the >50% loss reported by Lombardo et al. over 24 h [33] is therefore qualitative.

The FTIR comparison is based on a single averaged spectrum per material and state, uncorrected for baseline, so each coefficient carries no estimate of its own variability, and small localised differences, such as PA’s carbonyl-region residual, cannot be attributed with confidence. FTIR was not acquired on the SLS-processed PA12. Two further gaps should be distinguished. The biological qualification of the raw materials is the responsibility of their manufacturers and is documented in Table 1; we neither repeated nor verified it. What remains uncovered by that documentation, and unaddressed here, is the printed object itself: particulate release from a printed geometry, surface quality, and intraoral ageing were not assessed by us and are not within the scope of a raw-material certificate.

Priorities for future work are raster angle, infill density and print speed; print orientation; cyclic and long-duration loading in a simulated oral environment; sterilisation and ageing, which are known to alter the mechanical properties of printed and thermoformed aligners alike [34]; independent manufacturing batches; and biological evaluation. Coupling the data with finite-element models would allow the force delivered by a designed appliance to be predicted rather than inferred.

## 5. Conclusions

Under the thin-section conditions adopted, the six thermoplastic material–process systems differed markedly in apparent flexural stiffness and short-term stress relaxation. The PA12/SLS system was the stiffest but also relaxed the most; as the only laser-sintered material, its behaviour reflects both the polymer and the process, and the two cannot be separated here. In absolute terms, it still delivered 3.01 N after 10 min, exceeding the initial forces of PEEK, PETG, PA, and TPC, although not distinguishable from that of PC. PC and PEEK combined comparatively high stiffness with limited decay, with PC retaining 97.4% of its initial force. PETG was intermediate, and PA was substantially more compliant. TPC generated the lowest forces at the single 0.6 mm thickness tested; whether greater wall thickness would raise them into a useful range was not tested.

Expressed as the activation needed to reach clinically quoted force bands, PA12, PC, PEEK and PETG reach all three within sub-millimetre deflections. In contrast, PA and TPC reach only the intrusion band at this thickness, with TPC reaching it only in its upper part. Adjacent materials were frequently not statistically separable at *n* = 5, and where significance was reached, no claim of difference or of equivalence is made.

PETG, PC and PA are printable on accessible FDM equipment, while PEEK and PA12 require high-temperature or laser-sintering systems. These results provide specimen-level screening information for selecting material–process systems, and suggest which class of appliance each might serve. Still, they do not establish the suitability of any material for a finished appliance. Device-level testing, independent manufacturing batches, ageing and fatigue, dimensional, surface and optical assessment, and biological evaluation remain necessary before clinical use can be considered.

## Figures and Tables

**Table 1:** Materials used in the study, with polymer type, supplier, country of origin, and the biological documentation supplied by each manufacturer.

Commercial Name	Abbreviation/Polymer Type	Manufacturer/Supplier	Location	Biological Documentation Supplied by the Manufacturer
BIOFLEX^®^	TPC (thermoplastic copolyester elastomer)	FiloAlfa	Saint Peters, Italy	Tested to ISO 10993-4 [22] (haemocompatibility), ISO 10993-5 [23] (cytotoxicity) and ISO 10993-10 [24] (irritation and sensitisation); USP 32:2009 [25] Class VI.
NYLON 680™	PA (polyamide, nylon 6/69 copolymer)	Taulman3D	Linton, IN, USA	Manufactured in compliance with FDA 21 CFR 177.1500 (nylon resins) for food-contact use.
guidel!ne	PETG (polyethylene terephthalate glycol)	Taulman3 D	Linton, IN, USA	Base resin tested to ISO 10993 [21] for cytotoxicity, sensitisation, irritation, haemocompatibility and genotoxicity; USP Class VI.
OSSFILA^®^ Medical PEEK	PEEK (polyether-ether-ketone)	Novus Life Sciences	Hong Kong, China	Biocompatible and non-toxic to USP <88> Class VI; manufactured in an ISO 13485 [26]-certified cleanroom; sterilisable by gamma irradiation, e-beam, autoclave and ethylene oxide.
PC Medical	PC (polycarbonate)	Lattice Services	Orsay, France	Granules certified to ISO 10993-5 [23] (cytotoxicity) only, for skin contact; sterilisable by gamma irradiation, ethylene oxide and autoclave; not intended for implantable use.
PA 2201 (medical-grade PA12)	PA12 (polyamide 12)	Materialise	Leuven, Belgium	Certified biocompatible after testing for cytotoxicity, sensitisation, irritation, acute systemic toxicity and pyrogenicity.

**Table 2:** Printing parameters for each material are provided; common FDM settings are given in the text.

Material	Nozzle (°C)	Bed (°C)	Fan	Speed (mm/s)	Flow (mm^3^/s)	Drying (°C, h)
PETG (guidel!ne)	250	70/65	20%	40	6	60–65, 8
PC (PC Medical)	270	130	0%	25–30	4	80, 8
PA (Nylon 680)	255	55	0%	35–40	4	70–80, 8–12
PEEK (OSSFILA)	395	120	0%	15–20	–	–
TPC (BIOFLEX)	220	45	0%	20–25	2.5	60–70, 6–8
PA12 (PA 2201, SLS)	–	–	–	–	–	–

**Table 3:** Flexural parameters from the three-point bending test (mean ± 95% CI, *n* = 5). The apparent flexural modulus is the slope-based value over the 0–1 mm loading window, E_f_ = L^3^m/(4bh^3^).

Material	Flexural Modulus (MPa)	Flexural Stress @ 1 mm (MPa)	Total Energy (mJ)	Dissipated Energy (mJ)	Unloading Modulus (MPa)
PETG	509 ± 39	18.0 ± 1.6	1.13 ± 0.09	0.22 ± 0.05	502 ± 36
PC	750 ± 58	26.3 ± 2.0	1.61 ± 0.13	0.28 ± 0.04	737 ± 67
PA	85 ± 6	3.0 ± 0.3	0.19 ± 0.01	0.05 ± 0.01	86 ± 7
PEEK	687 ± 66	23.6 ± 2.1	1.35 ± 0.12	0.30 ± 0.05	658 ± 50
TPC	32 ± 2	1.2 ± 0.1	0.07 ± 0.01	0.00 ± 0.00	29 ± 2
PA12	989 ± 98	36.4 ± 3.5	2.24 ± 0.18	0.86 ± 0.13	924 ± 74

**Table 4:** Absolute force at the fixed 1 mm mid-span deflection, at the onset of the hold (F_0_) and after 10 min (F_10_), derived from the relaxation moduli (mean [95% CI], *n* = 5).

Material	F_0_ (N)	F_10_ (N)	Force Lost (N)	Force Retained (%)
PA12	3.87 [3.54–4.21]	3.01 [2.90–3.12]	0.86	77.9
PC	2.87 [2.59–3.16]	2.80 [2.52–3.08]	0.08	97.4
PEEK	2.55 [2.29–2.81]	2.43 [2.20–2.65]	0.12	95.3
PETG	2.03 [1.85–2.21]	1.83 [1.69–1.97]	0.20	90.2
PA	0.33 [0.30–0.36]	0.31 [0.29–0.34]	0.02	94.2
TPC	0.13 [0.12–0.14]	n.q.	n.q.	n.q.

**Table 5:** Mid-span deflection required for each material–process system to deliver the force associated with three classes of tooth movement, computed as δ = F/(0.00432 × E_f_) over the near-linear window. Values above 1 mm lie outside the range tested here and are extrapolations.

Material	Intrusion, 0.1–0.2 N	Tipping and Bodily Movement, 0.7–1.2 N	Rotation Control, 1.0–1.5 N
PA12	0.02–0.05 mm	0.16–0.28 mm	0.23–0.35 mm
PC	0.03–0.06 mm	0.22–0.37 mm	0.31–0.46 mm
PEEK	0.03–0.07 mm	0.24–0.40 mm	0.34–0.51 mm
PETG	0.05–0.09 mm	0.32–0.55 mm	0.45–0.68 mm
PA	0.27–0.54 mm	1.9–3.2 mm	2.7–4.1 mm
TPC	0.72–1.45 mm	5.0–8.6 mm	7.2–10.8 mm

## Data Availability

The raw data supporting the conclusions of this article will be made available by the authors on request.

## Supplementary Materials

The following supporting information can be downloaded at: https://www.mdpi.com/article/10.3390/dj14090565/s1, File S1: supplementary statistics.

## Abbreviations

The following abbreviations are used in this manuscript:

**Abbreviation**	**Definition**
ATR	Attenuated total reflectance
CI	Confidence interval
DLP	Digital light processing
FDM	Fused deposition modelling
FTIR	Fourier-transform infrared spectroscopy
IQR	Interquartile range
LCD	Liquid crystal display
PA	Polyamide
PA12	Polyamide 12
PC	Polycarbonate
PEEK	Polyether-ether-ketone
PETG	Polyethylene terephthalate glycol
SLA	Stereolithography
SLS	Selective laser sintering
TPC	Thermoplastic copolyester elastomer
